# Postnatal growth rate varies with latitude in range‐expanding geese: The role of plasticity and day length

**DOI:** 10.1111/1365-2656.13638

**Published:** 2021-11-28

**Authors:** Michiel P. Boom, Henk P. van der Jeugd, Boas Steffani, Bart A. Nolet, Kjell Larsson, Götz Eichhorn

**Affiliations:** ^1^ Vogeltrekstation—Dutch Centre for Avian Migration and Demography (NIOO‐KNAW) Wageningen The Netherlands; ^2^ Department of Animal Ecology Netherlands Institute of Ecology (NIOO‐KNAW) Wageningen The Netherlands; ^3^ Department of Theoretical and Computational Ecology Institute for Biodiversity and Ecosystem Dynamics University of Amsterdam Amsterdam The Netherlands; ^4^ Kalmar Maritime Academy Linnaeus University Kalmar Sweden

**Keywords:** adaptability, barnacle geese, environmental change, growth rate, migration, plasticity

## Abstract

1. The postnatal growth period is a crucial life stage, with potential lifelong effects on an animal's fitness. How fast animals grow depends on their life‐history strategy and rearing environment, and interspecific comparisons generally show higher growth rates at higher latitudes. However, to elucidate the mechanisms behind this gradient in growth rate, intraspecific comparisons are needed.
2. Recently, barnacle geese expanded their Arctic breeding range from the Russian Barents Sea coast southwards, and now also breed along the Baltic and North Sea coasts. Baltic breeders shortened their migration, while barnacle geese breeding along the North Sea stopped migrating entirely.
3. We collected cross‐sectional data on gosling tarsus length, head length and body mass, and constructed population‐specific growth curves to compare growth rates among three populations (Barents Sea, Baltic Sea and North Sea) spanning 17° in latitude.
4. Growth rate was faster at higher latitudes, and the gradient resembled the latitudinal gradient previously observed in an interspecific comparison of precocial species. Differences in day length among the three breeding regions could largely explain the observed differences in growth rate. In the Baltic, and especially in the Arctic population, growth rate was slower later in the season, most likely because of the stronger seasonal decline in food quality.
5. Our results suggest that differences in postnatal growth rate between the Arctic and temperate populations are mainly a plastic response to local environmental conditions. This plasticity can increase the individuals' ability to cope with annual variation in local conditions, but can also increase the potential to re‐distribute and adapt to new breeding environments.

## Graphical abstract

Over the past decades, Barnacle geese (*Branta leucopsis*) expanded there breeding range southwards from the Barents Sea coast in the Arctic, to the Baltic region and North Sea coast. The authors compared gosling growth in three populations spanning 17° in latitude, and show that goslings grow faster in the Arctic compared to temperate regions. The difference in growth rate is largely explained by differences in day length experienced by goslings at the different latitudes (varying from 16 h till continuous daylight). This highlights the importance of resource availability and indicates that growth rate is mainly a plastic response to local environmental conditions.

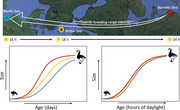

## 1 INTRODUCTION

The period of postnatal growth is a crucial stage in an animals' life cycle, with a clear link to fitness components such as reproduction and survival (Dmitriew, 2011; Haywood & Perrins, 1992; Starck & Ricklefs, 1998; Van der Jeugd & Larsson, 1998). The rate of growth is a basic life‐history trait, which is shaped by environmental conditions at the local breeding and rearing grounds as well as by an animal's life‐history strategy (Arendt, 1997; Dmitriew, 2011). According to life‐history theory, animals are expected to adapt growth rates to local conditions to maximize fitness. However, to cope with environmental variation, such as food availability and quality, growth rate also has to be flexible (Arendt, 1997; Dmitriew, 2011). Therefore, understanding which environmental factors influence growth rate and its flexibility is an important step in evaluating an animal's vulnerability and adaptability to environmental change.

In seasonal environments, the length of the growing season, which is negatively correlated with latitude, restricts the period available for growth. Correspondingly, a positive relationship between growth rate and latitude has been found in a variety of organisms, that is in fishes (Brown et al., 1998; Conover & Present, 1990), insects (Blanckenhorn et al., 2018; Kojima et al., 2020), amphibians (Lindgren & Laurila, 2005) and birds. The time constraint imposed by season length is especially pressing for Arctic‐breeding migratory birds, because offspring have to be fully developed and capable of leaving the breeding area before winter sets in (Alerstam & Hedenström, 1998; Owen & Black, 1989; Tomotani et al., 2016). By migrating between breeding and wintering areas, migratory birds are able to exploit seasonally occurring food peaks and avoid local food scarcity and harsh climatic conditions (Holt & Fryxell, 2011). Thus, performing migratory journeys is expected to enable fast growth, while also imposing the need to realize it.

Birds have been shown to benefit from fast growth since it shortens the period of vulnerability to size‐dependent predation (Dmitriew, 2011; Samelius & Alisauskas, 1999; Starck & Ricklefs, 1998). Furthermore, within bird populations, faster growth has been associated with larger adult size (Cooch et al., 1991; Larsson et al., 1998; Searcy et al., 2004; Van der Jeugd & Larsson, 1998) with fitness consequences throughout an individual's lifetime. However, growth itself might be costly. Fast growth can reduce resistance to starvation, increase cellular damage imposed by oxidative stress and reduce immune functioning, all of which may impact a bird's life span and functioning (Arendt, 1997; Dmitriew, 2011; Kim et al., 2011; Mangel & Munch, 2005). Among the environmental factors that control growth rates, food availability plays a central role. In birds, periods of food shortages have been shown to negatively affect muscle development and body mass increase (Killpack & Karasov, 2012) and, in strongly seasonal environments like the Arctic, a mismatch with the peak in food quality has been shown to result in slower growth (Brook et al., 2015; Ross et al., 2018).

The high productivity of the Arctic summer is an important prerequisite for successfully raising offspring in herbivorous and insectivorous species (Fokkema et al., 2020), and is considered a main driver of migration to the Arctic (Sedinger & Raveling, 1986). Furthermore, 24‐hr daylight during Arctic summers dramatically improves the potential feeding time of animals that rely on eyesight to forage (Schekkerman et al., 2003). Combined, the high productivity and unlimited feeding time in the Arctic result in high resource availability for Arctic‐breeding birds. In interspecific comparisons, higher growth rates have been reported for Arctic‐breeding waders such as red knot *Canutus canutus* and little stint *Calidris minuta* as compared to temperate‐breeding waders such as redshank *Tringa totanus*, lapwing *Vanellus vanellus* and black‐tailed godwit *Limosa limosa* (Schekkerman et al., 2003; Tjørve, 2007). The same pattern is found in altricial gulls and terns (*Larus* and *Sterna spec*.), where two populations of the same species (*Sterna paradisaea* and *Larus argentatus*) show a positive relation between latitude and growth rate (Klaassen, 1994; Tjørve, 2007). Although Schekkerman et al. (2003) mention the potential importance of day length and arthropod abundance for growing waders, the role of resource availability in explaining latitudinal differences in growth rate was not evaluated in detail in the aforementioned studies. Interspecific comparisons, like above, suffer from the fact that species are also bound to differ in other respects than breeding environment alone (Garland & Adolph, 1994). These limitations therefore call for studies using intraspecific comparisons across environments.

Here, we make a within‐species comparison of growth rates of barnacle goose goslings among three different populations (Barents Sea, Baltic Sea and North Sea) spanning 17° in latitude. These populations are genetically very similar and show substantial gene flow (Jonker et al., 2013). The Russian flyway population of barnacle geese has shown a strong increase over the past decades (over 7% annual increase since 1960; Madsen et al., 1999), and simultaneously expanded its traditionally Arctic breeding range by establishing new breeding colonies at stopover sites in the Baltic region and in the wintering area along the North Sea coast (Larsson et al., 1988; Van Der Jeugd et al., 2009). Barnacle geese breeding in the Baltic region shortened their migratory distance considerably compared to Arctic‐breeding geese, while barnacle geese breeding along the North Sea coast became sedentary. Besides differences in migratory strategy, geese from these populations also experience differences in their local breeding environments such as season length, day length and feeding conditions. Outside the breeding season, geese of all three populations share common wintering grounds along the North Sea coast. The rapid range expansion of the barnacle goose can be seen as a unique natural experiment, which allows to investigate how animals cope with new or changing environments by adopting new life‐history strategies. We relate the differences in growth rate to differences in environmental conditions at the breeding grounds and evaluate potential environmental constraints within the different populations. Furthermore, we assess whether differences in gosling growth among populations can be the result of microevolution or are to be attributed to developmental phenotypic plasticity (i.e. the ability of an individual to adapt to novel circumstances through flexible expression of a trait; Dobzhansky, 1970). Finally, we compare the latitudinal gradient in growth rates observed in barnacle geese to the latitudinal gradients observed in precocial waterfowl and waders based on previously published growth rates.

## 2 MATERIALS AND METHODS

### 2.1 Data collection

We collected biometric data on growing goslings during long‐term studies in colonies from three study populations (Figure S1): (a) A long‐distance migratory population breeding in the Arctic in Kolokolkova Bay along the Barents Sea coast (68°35′N, 52°20′E), data collected in 6 years between 2003 and 2015; (b) A short‐distance migratory population breeding on Gotland in the Baltic Sea (57°25′N, 18°53′E) data collected in 15 years between 1986 and 2000; (c) A sedentary population breeding in the Netherlands along the North Sea (51°40′N, 4°14′E) data collected in 5 years between 2004 and 2018 (Eichhorn et al., 2010; Larsson et al., 1988; Van der Jeugd et al., 2003, 2009).

Our analysis is based on all measured goslings with known age (see Supporting Information for age determination methods; Sample sizes: Barents Sea = 392; Baltic Sea = 933; North Sea = 116; Table S1). Sex was determined based on cloacal inspection. Goslings were weighed in a bag using a Pesola spring scale with an accuracy of ±5 g (if <600 g) or a digital hand scale or Pesola spring scale with an accuracy of ±10 g (if >600 g). A calliper (±0.1 mm) was used to measure the outer length of the bent tarsus. Head length was measured using a ruler (±1 mm). Body mass and body size are correlated (Figure S2), but are generally analysed separately when modelling growth (Starck & Ricklefs, 1998; Tjørve & Tjørve, 2010). In the North Sea and in the Barents Sea population, 99 and 26 goslings, respectively, were measured immediately upon hatch to estimate hatchling size. No initial size measures upon hatching were taken in the Baltic Sea population. Median age (and range) of all goslings of known age was 29 (5–42) days (Barents Sea), 46 (28–63) days (Baltic Sea) and 44 (10–86) days (North Sea) for the three colonies, respectively. Catching, ringing and measuring of geese were done under permits issued by the Dutch Ministry of Agriculture, Nature and Fisheries (permit: no. 951 ‘vogelwet 1936 & jachtwet’), the Swedish Museum of Natural History (permits: 523 and 523M001, issued to K. Larsson and H. van der Jeugd) and the Ministry of Natural Resources and Forestry Arkhangelsk (permits: 204‐08/1549, 204‐08/2125). Additionally, permission was obtained from landowning organizations: Staatsbosbeheer and County Administration Gotland.

### 2.2 Growth models

We modelled gosling growth separately for male and female goslings using a Gompertz model (Gompertz, 1825), which is commonly used for precocial species (Schekkerman et al., 1998; Tjørve & Tjørve, 2010, 2017), with a fixed initial value (hatching size) as proposed by Tjørve and Tjørve (2017), using the following formula:

(1) $$\text{Size}=\frac{A}{e^{\log\left(\frac{A}{I}\right)}\times e^{\left(‐k\times \text{age}\right)}}.$$

In this formula, biometric size (head length in mm, tarsus length in mm or body mass in g) is modelled as a function of age (in days). We chose to use the Gompertz equation, because it has been applied successfully to waterfowl data in the past and allows for easy comparison with other studies (Sedinger, 1986). In the equation, *A* represents the asymptote, which has been fixed to the average adult size for males and females (Table S2), respectively, as suggested by Austin et al. (2011). Asymptotic values were based on measurements of adult geese caught during moult in the three study areas. Because adult size and body mass were similar for all three populations (see Supporting Information), we used adult size and body mass averaged over all three populations. The size at hatch (when age = 0) is given by *I*, which is calculated based on measurements taken immediately upon hatch. It replaces the inflection point parameter in the original Gompertz function (Tjørve & Tjørve, 2017). We used the same value for *I* in models for males and females of all three populations, since we were not able to distinguish between males and females at day 0. Differences in average hatching size were smaller than 1 g or 1 mm between the Barents Sea and North Sea populations, so we used the same averages for all three populations. Parameter *k* represents the growth coefficient, and is estimated by the model. In the (few) cases of multiple recaptures of an individual, only data from the first capture were used in our analyses to avoid potential bias in recapture data due to repeated handling stress. Nest (brood) ID was included as a random effect on *k* to account for statistical dependence due to genetic background, similar rearing environment and potential maternal effects (Sofaer et al., 2013). Since data were collected over multiple years, we also included random cohort effects to account for variation caused by annual differences in phenology and growing conditions. We nested the random effect of nest within the effect of cohort:

(2) $$\text{Size}=\frac{A}{e^{\log\left(\frac{A}{I}\right)}\times e^{\left(\left(‐k+ki+kij\right)\times \text{age}\right)}},$$

where *k_i_* represents the random cohort effect and *k_ij_* the random nest effect. Random nest and cohort effects and their respective errors were expected to be normally distributed with a mean of zero. Growth models were constructed using a nonlinear mixed effects model approach, using the ‘nlme’ package in r (Pinheiro et al., 2012; R Development Core Team, 2010).

### 2.3 Comparing populations

Specific testing of differences in gosling growth rate between populations was done by adding dummy variables for the three populations to expression (2) as proposed by Sofaer et al. (2013).

(3) $$\text{Size}=\frac{A}{e^{\log\left(\frac{A}{I}\right)}\times e^{\left(\left(\left(‐k+ki+kij\right)+k_{\text{Ba}}\times P_{\text{Ba}}+k_{\text{NS}}\times P_{\text{NS}}\right)\times \text{age}\right)}}.$$

Here, *P*_Ba_ and *P*_NS_ are the dummy variables for the Baltic and North Sea population (represented by 1 or 0) and *k*_Ba_ and *k*_NS_ are the population‐specific differences to the *k* of the Barents Sea population. In this way, we could determine parameter estimates describing the difference in growth rate among populations. We constructed separate models for male and female goslings since the sexes have different asymptotes.

### 2.4 Day length

The number of daylight hours that had accumulated between hatching and capture was calculated for each gosling. Daylight was determined as the period between dawn and dusk, and was calculated based on the coordinates of the three breeding colonies using the r package ‘Suncalc’ (Thieurmel & Elmarhraoui, 2019). To model biometric size as a function of daylight hours, we used the same formula as expression (3), replacing ‘age in days’ by ‘daylight hours’ experienced by each individual gosling.

In addition to our analysis with fixed population effects included in a random Gompertz model (following Sofaer et al., 2013), we analysed our data using GLMMs on the residuals of non‐random Gompertz models for males and females (see Supporting Information).

### 2.5 Effect of hatch date on growth

The effect of hatch date on growth was analysed using the residuals of the non‐random Gompertz models (expression (1)), hereafter referred to as ‘residual head length’, ‘residual tarsus length’ and ‘residual body mass’. The residuals were calculated by subtracting the expected value of morphometric size of a gosling at a certain age based on the fitted growth curve from the observed size. Residuals of males and females were analysed collectively. We calculated relative hatch dates by centralizing hatch dates within each cohort, because years can differ in onset of spring and consequently in timing of breeding and hatching. For the calculation of the relative hatch date for each gosling, we used the mean hatch date of the colonies (not only of the recaptured goslings), as established from nest monitoring (see Supporting Information for details). We constructed GLMMs with fixed effects for population, hatch date and their interaction. Sex was added as fixed effect to account for potential differences between the residuals of models for males and females. NestID and cohort were included as random effects, with nestID nested in cohort. We used a backward selection procedure using Akaike information criterion (AIC) to exclude factors that did not contribute to the fit of the model.

### 2.6 Phenotypic plasticity or evolutionary response

To investigate whether any observed differences in growth rate among the study populations can be the result of microevolution or have to be (partly) attributed to phenotypic plasticity, we expressed the rate of change in haldanes (Gienapp et al., 2007). The haldane expresses the rate of change per generation in phenotypic standard deviations (*SD*) and is calculated with the formula given in expression (4).

(4) $$h=\frac{\frac{X_{2}}{S_{p}}‐\frac{X_{1}}{S_{p}}}{g}.$$

Here, *h* represents the phenotypic change in haldanes, *X*_1_ and *X*_2_ are the trait mean values of two populations (synchronic comparison), *S*_p_ is the pooled standard deviation from *X*_1_ and *X*_2_, and *g* is the number of generations (Hendry & Kinnison, 1999).

We used the Gompertz growth rate of the Barents Sea and North Sea population for *X*_1_ and *X*_2_, since these two populations are expected to represent the largest difference. *S*_p_ is calculated using the standard deviations estimated by the growth models. The number of generations is calculated based on a generation time for barnacle geese of 7.5 years (Dillingham, 2010), and a period of change of 30 years (period from 1985 till 2015 in which the establishment of the North Sea barnacle goose colony took place).

## 3 RESULTS

### 3.1 Population comparisons

**FIGURE 1:**
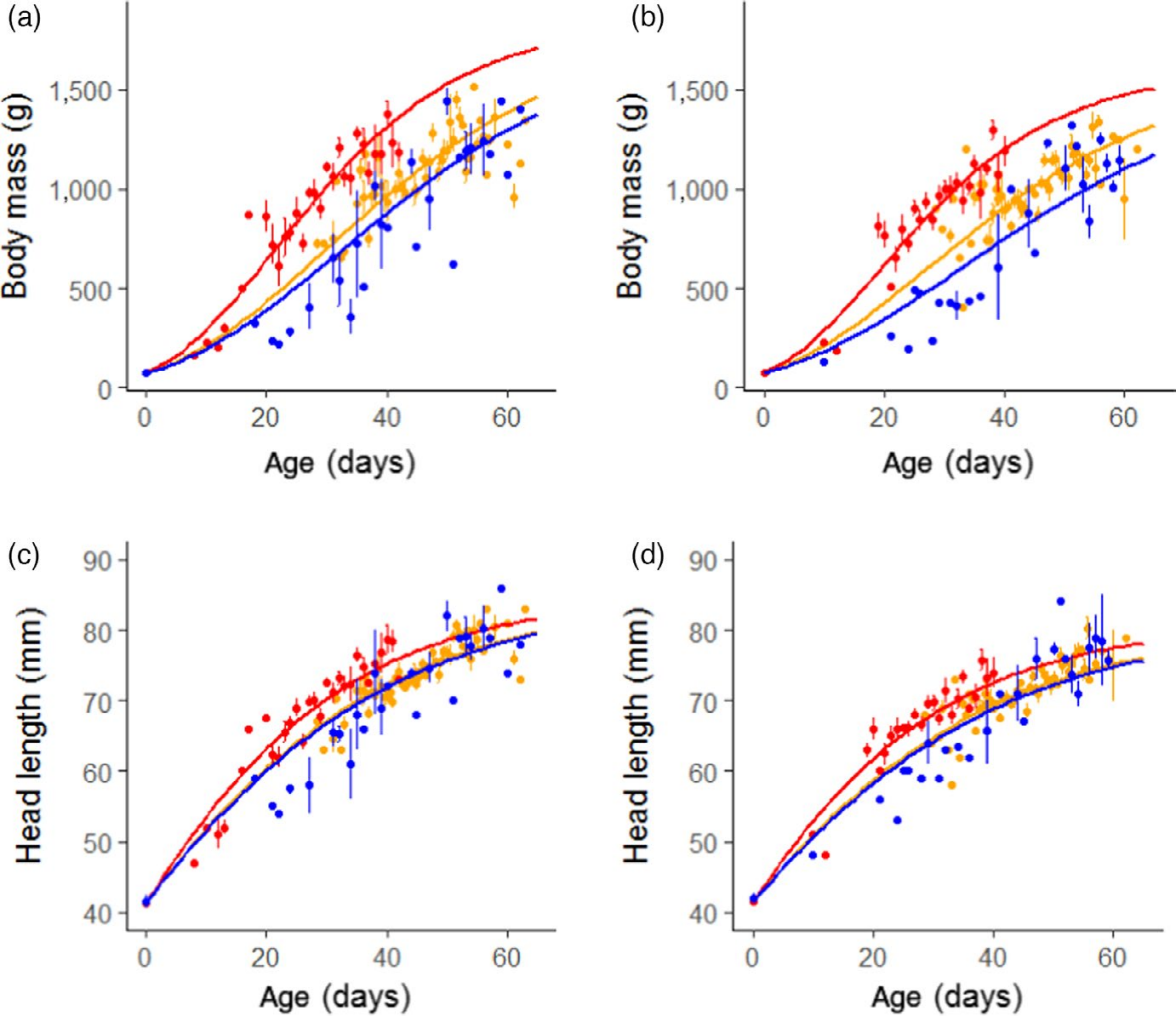
Gompertz growth models for body mass and head length in relation to age in days. Growth models for males are shown in panels (a) and (c); for females in panels (b) and (d). The Barents Sea population is shown in red, the Baltic population in yellow and the North Sea population in blue. Data points show daily means ± *SE*

The growth rate of gosling body mass, head length and tarsus length were found to differ among the three populations studied (Figure 1, Figure S3A,B, Table 1).

**TABLE 1:** Overview of the estimated growth coefficients (*k*) by the Gompertz growth models with age in days, and age in hours of daylight experienced. Estimates are given for body mass, head length and tarsus length for males and females of all three study populations separately. Values give the estimate ± *SE*. Letters indicate significant differences between populations (see main text for test statistics). Note that populations are compared within sexes; thus, letters only indicate differences among populations within sex

		Body mass		Head length		Tarsus length	
		Age (days)	Age (daylight)	Age (days)	Age (daylight)	Age (days)	Age (daylight)
Barents Sea	Male	0.056 ± 0.0021^a^	0.0023 ± 0.00010^q^	0.044 ± 0.0019^a^	0.0019 ± 0.00009^q^	0.087 ± 0.0056^a^	0.0036 ± 0.00047^q^
Baltic Sea	Male	0.040 ± 0.0013^b^	0.0020 ± 0.00006^r^	0.037 ± 0.0012^b^	0.0019 ± 0.00006^q^	0.078 ± 0.0037^ab^	0.0044 ± 0.00034^q^
North Sea	Male	0.036 ± 0.0024^b^	0.0020 ± 0.00011^r^	0.036 ± 0.0026^b^	0.0020 ± 0.00011^q^	0.064 ± 0.0068^b^	0.0035 ± 0.00052^q^
Barents Sea	Female	0.059 ± 0.0020^a^	0.0025 ± 0.00010^q^	0.045 ± 0.0020^a^	0.0019 ± 0.00009^q^	0.089 ± 0.0054^a^	0.0037 ± 0.00032^qr^
Baltic Sea	Female	0.042 ± 0.0012^b^	0.0021 ± 0.00006^r^	0.038 ± 0.0012^b^	0.0019 ± 0.00006^q^	0.074 ± 0.0034^b^	0.0041 ± 0.00024^r^
North Sea	Female	0.035 ± 0.0025^c^	0.0019 ± 0.00012^r^	0.035 ± 0.0023^b^	0.0020 ± 0.00012^q^	0.059 ± 0.0066^c^	0.0031 ± 0.00039^q^

In both males and females, body mass growth in the Barents Sea population was faster than in the Baltic population (Males: *t*_192_ = −6.54, *p* < 0.001; Females: *t*_187_ = −7.00, *p* < 0.001) and North Sea population (Males: *t*_192_ = −6.09, *p* < 0.001; Females: *t*_187_ = −7.43, *p* < 0.001). No significant difference in body mass growth was found between males in the Baltic and North Sea population (*t*_192_ = −1.27, *p* = 0.20), but the difference was significant for females (*t*_187_ = −2.60, *p* < 0.05).

A similar pattern is observed for the growth rate of head length. Significantly faster growth was observed in the Barents Sea population than in the Baltic population (Males: *t*_194_ = −3.07, *p* < 0.01; Females: *t*_187_ = −3.73, *p* < 0.001) and North Sea population (Males: *t*_194_ = −2.62, *p* < 0.01; Females: *t*_187_ = −3.13, *p* < 0.01). The difference between goslings in the Baltic and North Sea populations was not significant for either sex (Males: *t*_194_ = −0.35, *p* = 0.72; Females: *t*_187_ = −0.45, *p* = 0.65).

Tarsus growth of female goslings (Figure S3A,B) was also significantly faster in the Barents Sea population than in the Baltic population (*t*_187_ = −2.34, *p* < 0.05) and North Sea population (*t*_187_ = −3.51, *p* < 0.001). In males, the tarsus growth rate only differed between the Barents Sea and the North Sea population (*t*_196_ = −2.64, *p* < 0.01). No significant difference between males in the Baltic and North Sea populations was observed (*t*_196_ = −1.77, *p* = 0.08), but females differed significantly (*t*_187_ = −2.02, *p* < 0.05).

The populations differences reported above are supported by the GLMM analysis on the residuals of the non‐random Gompertz models. The models that included ‘population’ had consistently lower AICc values than models that did not include ‘population’ (Table S3).

### 3.2 Day length

**FIGURE 2:**
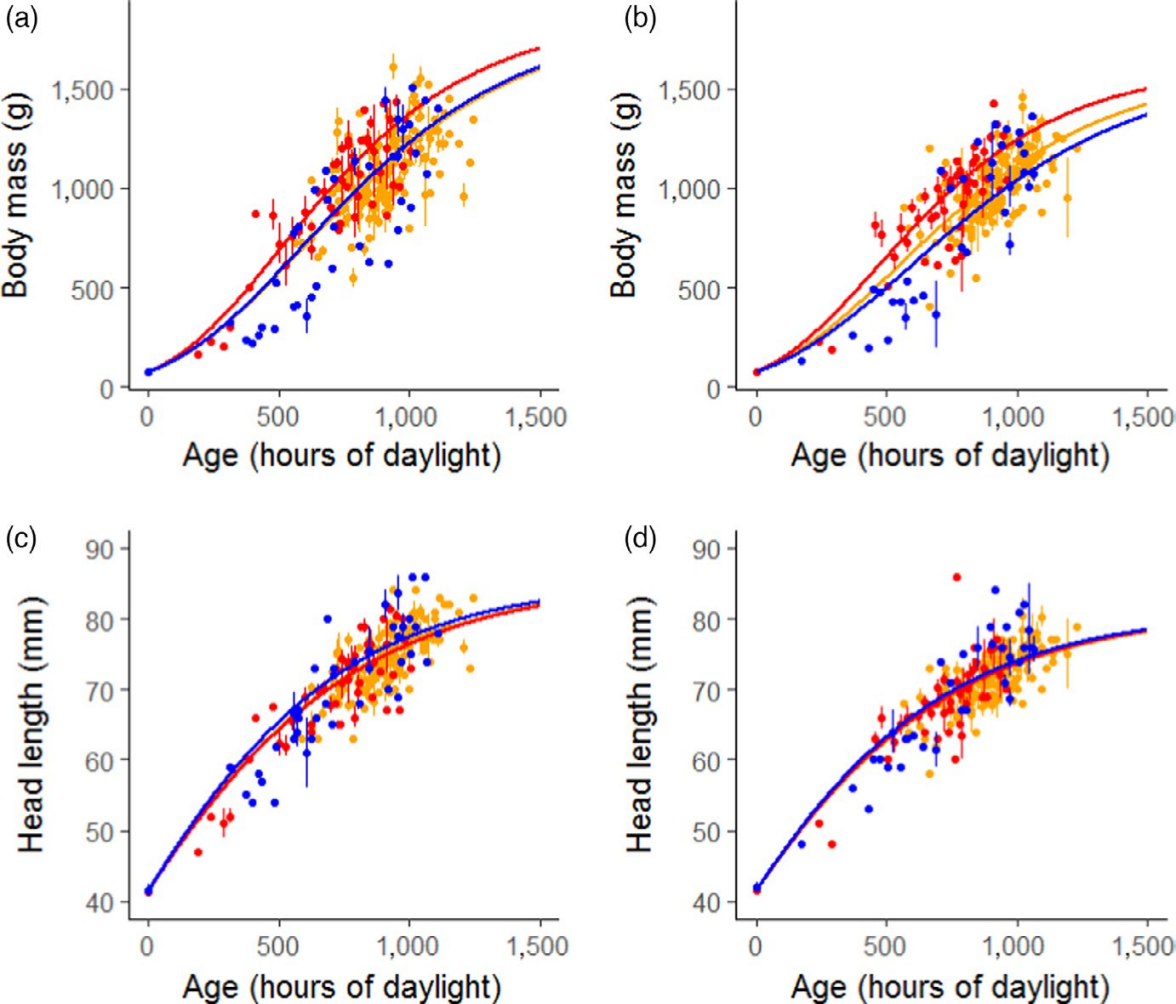
Gompertz growth models for body mass and head length in relation to hours of daylight experienced by each gosling. Growth models for males are shown in panels (a) and (c); for females in panels (b) and (d). The Barents Sea population is shown in red, the Baltic population in yellow and the North Sea population in blue. Data points show daily means ± *SE*

Differences in gosling growth rate among populations were largely explained by differences in day length (Figure 2, Table 1). When gosling age (in days) is replaced by accumulated daylight experienced since hatch (in hours), we found no difference in gosling growth rate among the three populations for head length. For tarsus growth (Figure S3C,D), most differences were no longer significant, except for the difference between females of the Baltic Sea and North Sea populations (*t*_187_ = −2.34, *p* < 0.05). For body mass, differences in gosling growth rate between the Baltic Sea and North Sea populations were no longer significant, while the difference between the Barents Sea population on the one hand and the Baltic and North Sea population on the other hand was reduced, but persisted for both females (Barents Sea vs. Baltic: *t*_187_ = −3.02, *p* < 0.01; Barents Sea vs. North Sea: *t*_187_ = −3.35, *p* < 0.01) and males (Barents Sea vs. Baltic: *t*_192_ = −2.94, *p* < 0.01; Barents Sea vs. North Sea: *t*_192_ = −2.06, *p* < 0.05).

These results were in line with the GLMM analysis on the residuals of the non‐random Gompertz models. When comparing the AICc values of the models with ‘daylight experienced since hatch’, ‘population’ was not retained in the most parsimonious models for head length and tarsus length in both males and females. For body mass, ‘population’ was retained in the most parsimonious model for both sexes (Table S3).

### 3.3 Effects of hatch date

**FIGURE 3:**
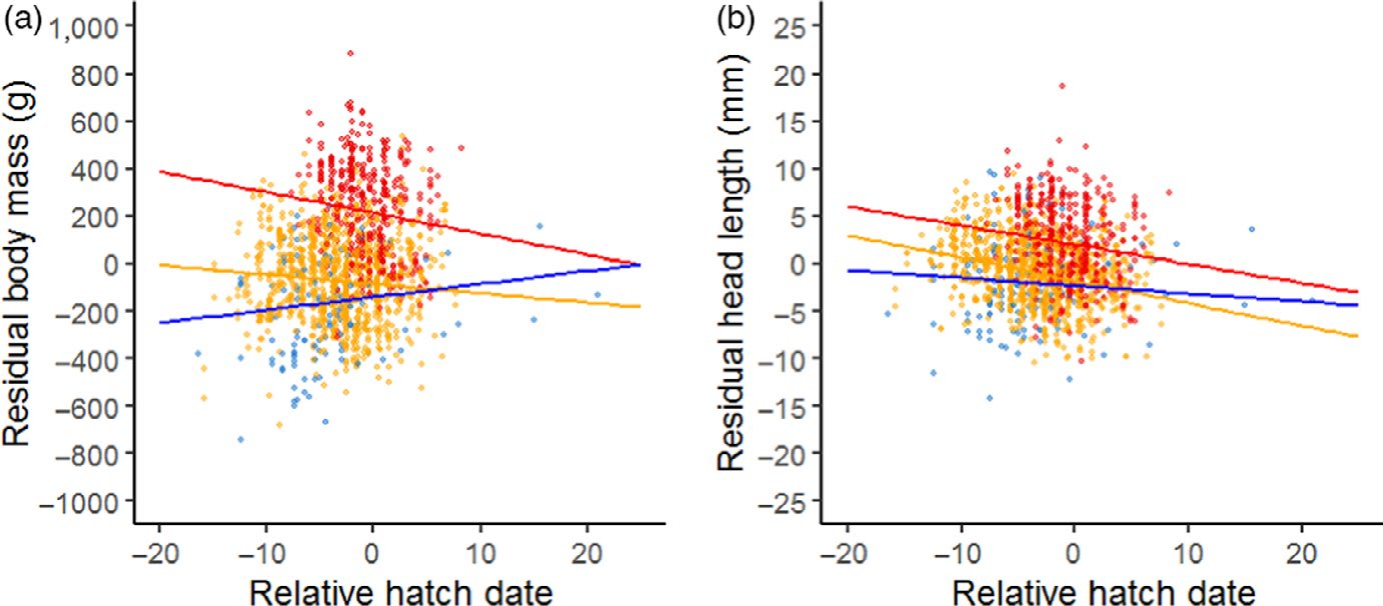
The relationship between relative hatch date and residual body mass (panel a) and residual head length (panel b) extracted from the non‐random Gompertz growth models. The Barents Sea population is shown in red, the Baltic population in yellow and the North Sea population in blue. Data points show individual residuals, lines indicate the results of the GLMM. Note that the slopes for residual head length are not found to be significantly different among populations

We found a significant interaction effect of population and relative hatch date on the residual body mass (*F*_2, 634_ = 4.56, *p* < 0.05; Figure 3a). The effect of relative hatch date was negative in the Barents Sea and Baltic Sea population (−8.85 ± 4.52 and −3.91 ± 1.78), that is, late‐hatched goslings grew slower and were therefore relatively smaller, while no effect of hatch date was found in the North Sea population (5.39 ± 3.64).

The interaction effect of relative hatch date and population on residual head length was retained in the model, but was not significant (*F*_2, 639_ = 2.76, *p* = 0.064; Figure 3b). The pattern was similar to the pattern found for body mass, that is, later hatched goslings tended to grow slower in the Barents Sea and Baltic Sea population, while this trend was absent in the North Sea population.

The interaction effect of relative hatch date and population was excluded from the model on residual tarsus length. However, the best model did contain relative hatch date as independent variable, showing a significant negative effect (*F*_1, 641_ = 37.09, *p* < 0.001; Figure S4).

### 3.4 Phenotypic plasticity versus micro‐evolution

The differences in growth rate between the Barents Sea and North Sea population, expressed in haldanes, ranged between 0.08 and 0.24 for all three biometric measures (Table 2). In general, the supposed change in standard deviation units per generation was larger in females compared to males. In both males and females, the same pattern was observed, with the largest change per generation observed in body mass growth rate (0.18 and 0.24 *SD* units per generation, respectively).

**TABLE 2:** Calculated haldanes for the differences in growth coefficients between the Barents Sea and North Sea populations. Generation time for the calculations was 7.5 years, and the period of change was 30 years

Biometric measure	Males	Females
Body mass	0.176	0.236
Head length	0.077	0.100
Tarsus length	0.077	0.113

## 4 DISCUSSION

This study revealed clear differences in postnatal growth rate of head length, tarsus length and body mass of barnacle geese raised along a latitudinal gradient from the Arctic to the temperate zone. In the Arctic, goslings experienced the highest growth rates for all studied morphological measurements. At the North Sea, growth rates were lowest, while goslings in the Baltic population showed intermediate values, which were closer to the growth rates found in the North Sea than in the Arctic population. By including three populations of the same species along a latitudinal gradient, our results are more robust compared to previous studies based on interspecific comparisons.

### 4.1 Intraspecific and interspecific patterns of growth rate with latitude

**FIGURE 4:**
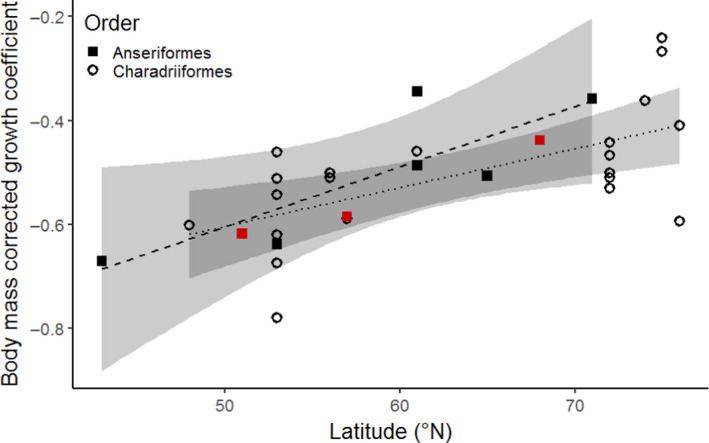
Relationship between latitude and Gompertz growth coefficients in precocial species in the Northern hemisphere. The growth coefficient is corrected for the LOG(body mass) to make species of different sizes comparable. Growth coefficients of Anseriformes are given by filled squares (see Table S4 for references). Red squares represent the growth coefficients of our barnacle goose study populations. Growth coefficients of Charadriiformes (open circles) have been retrieved from Tjørve (2007) and Tulp (1998). Regression lines are given for Anseriformes, without the barnacle goose populations (dashed line; *y* = 0.012*x* − 1.18) and Charadriiformes (dotted line; *y* = 0.0064*x* − 0.90) species separately. Shaded bands represent the 95% confidence interval

The intraspecific growth rate differences we found are in line with interspecific differences found among gulls, terns and waders, which also show an increase in chick growth rate with increasing breeding latitude (Klaassen, 1994; Schekkerman et al., 2003; Tjørve, 2007; Tjørve et al., 2009). When we limit our comparison of growth coefficients along a latitudinal gradient to precocial species (Table S4), because altricial species are known to grow faster (Starck & Ricklefs, 1998), and correct the growth rate for the Log(body mass) of each species, respectively, since growth rate scales with body mass (Tjørve, 2007), we find that the intraspecific linear increase in growth rate with latitude for barnacle geese is similar to the interspecific pattern in Charadriiformes and other Anseriformes (Figure 4). Furthermore, this pattern holds regardless of foraging guild (wader chicks being insectivorous and waterfowl being herbivorous), confirming findings of Tjørve (2007) for waders and gulls.

### 4.2 Resource availability and phenotypic plasticity

Our results suggest that the observed differences in growth rate are mainly the result of differences in resource availability. Continuous daylight during the Arctic summer increases potential feeding time for Arctic‐breeding geese with approximately 8 hr (33%) compared to feeding time for temperate breeding geese, and with approximately 2 hr (10%) for the Baltic Sea population. Barnacle geese make use of the extended day length by adapting their circadian rhythm (Eichhorn et al., 2021). The effect of daylight on growth has extensively been shown in poultry, where increasing day length led to increased food consumption resulting in higher growth rates (Kleinpeter & Mixner, 1947; Wineland, 2002). This increased food intake is expected to require a larger metabolic machinery. Correspondingly, Eichhorn et al.(2019) report a higher resting metabolic rate in goslings from the Barents Sea than in those from the North Sea population. Indeed, our results show that correcting for increasing day length with latitude largely explains growth rate differences among the three study populations, in particular for the structural size measures. Some differences in body mass growth rate persist after correcting for day length, with Arctic goslings still gaining body mass faster than goslings from both temperate breeding populations. This rapid body mass growth after correcting for the longer daylight regime most likely results from the distinct peak in herbivorous food quality experienced by goslings in the Arctic (Van der Graaf et al., 2006; Van der Jeugd et al., 2009).

In order for goslings to benefit from this food peak, timing of reproduction is essential (Lameris et al., 2017; Nolet et al., 2020; Van der Graaf et al., 2006; Van der Jeugd et al., 2009), as is illustrated by the negative effect of hatch date on residual head length and tarsus length, and its interaction with population. We found the strongest negative effect of hatch date on residual body mass in the Barents Sea population, a weaker but still significant negative effect in the Baltic Sea population, and no effect of hatch date in the North Sea population. Although differences among populations were not significant, the patterns showed a relationship similar to that in body mass. While the impact of hatch date on growth rate has been shown before in Arctic geese (Cooch, 2002; Doiron et al., 2015; Gauthier et al., 2006; Sedinger & Flint, 1991), we show here that this impact increases with latitude and is absent in the sedentary temperate population. Lindholm et al. (1994) showed experimentally that the decrease in growth rate of later‐hatched goslings in the Arctic is mainly the result of a decrease in forage quality. While birds in the Barents Sea population are generally able to utilize the food quality peak, hatching in the North Sea and Baltic population occurs too late (Van der Jeugd et al., 2009). Hence, the food peak may not only be higher in the Arctic than in temperate areas, but breeding may also be better timed so goslings can profit from it. Larsson and Forslund (1991) showed that differences in food quality not only affect the growth of barnacle goose goslings but also their final adult size. Similar results were found in lesser snow geese *Anser caerulescens caerulescens* and black brants *Branta bernicla nigrans* (Cooch et al., 1991, 1996; Sedinger & Flint, 1991). This developmental plasticity itself is adaptive, as it allows a growing individual important leeway when environmental conditions limit growth and the programmed size is out of reach.

Although microevolution can be fast under strong selection (Endler, 1980), especially when strong founder effects occur (Grant & Grant, 1995), the differences in growth rate observed between the Arctic and North Sea population appear too high to be caused by micro‐evolution alone, in the relatively short time span between the time of establishment of the new populations and the time of our measurements. Only the smallest difference in growth rate between both populations (male head length and tarsus length) remained within the limits of plausible microevolution, with 0.077 haldanes still being higher than 75% of 2,414 evolutionary rates reported by Hendry et al. (2008). With values over 0.10 haldanes (for differences in body mass and female head and tarsus length) being higher than 97% of the evolutionary rates reported by Hendry et al. (2008), plasticity appears to be the main mechanism behind the observed differences in growth rate.

Although our results indicate a prominent role for phenotypic plasticity in response to resource availability, it is not possible to fully disentangle the contributions of plasticity and selection. The higher growth rate found in the Barents Sea population might be the result of within season selection for faster growth, because smaller goslings may be more likely to die before recapture due to predation and adverse weather conditions. Additionally, the faster body mass growth in the Barents Sea population, even after correcting for the effect of daylight, might reflect selection for faster growth to ensure goslings fledge in time to escape harsh weather conditions with the autumn migration. Larger, faster growing goslings have higher survival prospects on their first autumn migration (Loonen et al., 1999). Van der Jeugd et al. (2009) reported a much steeper decrease in post‐fledging survival with hatch date in the Barents Sea population as compared to the Baltic and North Sea population, indicating that the effect of season length and hatch date is much more prominent in the Arctic. Timing of breeding likely correlates with resource availability for goslings, because hatching early ensures goslings are better able to utilize the food quality peak. Therefore, it is possible that selection occurs on timing of breeding, whereas gosling growth rate remains a plastic response to resource availability. This is also supported by our findings of a stronger negative relation between hatch date and growth rate with latitude. The narrow food peak in the Arctic would result in a stronger negative effect of hatching too late. Consequently, individual timing of breeding in the Barents Sea population is more concentrated, with 90% of the nests being initiated within a period of 12 days, compared to 15 days in the Baltic Sea population and 36 days in the North Sea population (Van der Jeugd et al., 2009), again illustrating the stronger time constraints on reproduction in the Arctic.

In this study, we used the Gompertz equation to construct growth curves for our study populations. While there are other, more flexible equations to model growth, like the Richards‐equation, using the exact same equation (Gompertz) for each population allows for a comparison among study populations, as well as a comparison with growth coefficients published for other species.

In the absence of differences in adult size among the study populations, we used one common asymptote for each of the three study populations, instead of estimating these with the model. While using fixed asymptotes might lead to a lower fit of the model, we follow Austin et al. (2011) who argue that when adult size is known, fixing asymptotes is desirable to get biologically meaningful growth estimates. The absence of consistent variation in adult body size despite the differences in growth rate that we found here may mean that these differences are offset by differences in the length of the growing season. While growing slowly, temperate goslings have more time to reach adult body size compared to Arctic goslings, since they do not leave the breeding grounds to migrate south. Furthermore, variation in adult body size in different cohorts within the same population caused by annual differences in food quality can be large (Larsson & Forslund, 1991), and may obscure potential population differences. By including random effects for cohort, we corrected for variation in growing conditions among years. To account for a trend in body size over the study period as a result of an increasing mismatch with the peak in food availability due to climate change (Doiron et al., 2015; Nolet et al., 2020), we checked the residuals of the non‐random Gompertz curves for the Baltic Sea population, which has the best data coverage over time. We found no trend for the residuals of head length and tarsus length, but found a negative trend for the residuals of body mass, although its effect was small (−5.6 g/year decrease), corresponding with a decrease of 87 g over the 15‐year study period (around 5% of the mean asymptotic body mass). Unfortunately, data coverage in the Barents Sea and North Sea population is not (yet) adequate to check whether this may be a general effect that can indeed be attributed to climate change but this may be confirmed in a later study.

## 5 CONCLUSIONS

Our results show that goslings from an Arctic (migratory) population grow faster than goslings from a temperate (non‐migratory) population, while goslings raised at an intermediate latitude show intermediate growth rates. Our analysis suggests that these differences are caused by a plastic response to local environmental conditions such as day length and food quality. However, it is not possible to fully disentangle these effects from micro‐evolutionary adaptations of growth rate to latitude without doing experimental studies. One way to bring this further is to set up a ‘common garden’ and study the growth of goslings from different breeding populations under the same rearing conditions.

The differences we show in growth rates of goslings in Arctic and temperate populations of the same species help unravel the costs and benefits of a migratory lifestyle. The costs involved in completing a migratory journey should be balanced by fitness benefits. Changing conditions in both the Arctic and temperate zone can influence the cost–benefit balance of a migratory journey. In their temperate wintering and breeding sites, barnacle geese profit from managed grasslands that provide a diet of improved food quality (Eichhorn et al., 2012). Climate warming, on the other hand, pushes migratory geese to their limit to arrive at their Arctic breeding grounds in time to ensure their goslings can profit from the food peak (Lameris et al., 2018). Under these developments, the benefits of migration might not outweigh the costs any longer, whereas the costs of breeding in temperate areas may not be as high as they used to be. Plasticity in growth rates can be an important factor enabling species to be flexible enough to adapt to new or rapidly changing breeding environments.

## CONFLICT OF INTEREST

The authors have no conflict of interest to declare.

## AUTHORS' CONTRIBUTIONS

G.E. conceived the study; G.E., H.P.v.d.J., K.L., B.S., M.P.B. collected the data; M.P.B. analysed the data; M.P.B., H.P.v.d.J., B.A.N. and G.E. contributed to the interpretation of the data and results; M.P.B. led the writing of the manuscript. All authors contributed critically to the drafts and gave final approval for publication.

## Supporting information

Supplementary Material

## Data Availability

Data available from the Dryad Digital Repository https://doi.org/10.5061/dryad.qjq2bvqhc (Boom et al., 2021).

## References

[jane13638-bib-0001] Alerstam, T. , & Hedenström, A. (1998). The development of bird migration theory. Journal of Avian Biology, 29(4), 343. 10.2307/3677155

[jane13638-bib-0002] Arendt, J. D. (1997). Adaptive intrinsic growth rates: An integration across taxa. The Quarterly Review of Biology, 72(2), 149–177. 10.1086/419764

[jane13638-bib-0003] Austin, S. H. , Robinson, T. R. , Robinson, W. D. , & Ricklefs, R. E. (2011). Potential biases in estimating the rate parameter of sigmoid growth functions. Methods in Ecology and Evolution, 2(1), 43–51. 10.1111/j.2041-210X.2010.00055.x

[jane13638-bib-0004] Blanckenhorn, W. U. , Bauerfeind, S. S. , Berger, D. , Davidowitz, G. , Fox, C. W. , Guillaume, F. , Nakamura, S. , Nishimura, K. , Sasaki, H. , Stillwell, R. C. , Tachi, T. , & Schäfer, M. A. (2018). Life history traits, but not body size, vary systematically along latitudinal gradients on three continents in the widespread yellow dung fly. Ecography, 41(12), 2080–2091. 10.1111/ecog.03752

[jane13638-bib-0005] Boom, M. P. , Van der Jeugd, H. P. , Steffani, B. , Nolet, B. A. , Larsson, K. , & Eichhorn, G. (2021). Data from: Postnatal growth rate varies with latitude in range‐expanding geese: The role of plasticity and day length. Dryad Digital Repository, 10.5061/dryad.qjq2bvqhc PMC9300058 34807466

[jane13638-bib-0006] Brook, R. W. , Leafloor, J. O. , Abraham, K. F. , Douglas, D. C. , Brook, R. W. , Leafloor, J. O. , Abraham, K. F. , & Douglas, D. C. (2015). Density dependence and phenological mismatch: Consequences for growth and survival of sub‐arctic nesting Canada Geese. Avian Conservation and Ecology, 10(1), 1–11. 10.5751/ACE-00708-100101

[jane13638-bib-0007] Brown, J. J. , Ehtisham, A. , & Conover, D. O. (1998). Variation in larval growth rate among Striped Bass stocks from different latitudes. Transactions of the American Fisheries Society, 127(4), 598–610. 10.1577/1548-8659(1998)127<0598:vilgra>2.0.co;2

[jane13638-bib-0008] Conover, D. O. , & Present, T. M. C. (1990). Countergradient variation in growth rate: Compensation for length of the growing season among Atlantic silversides from different latitudes. Oecologia, 83(3), 316–324. 10.1007/BF00317554 28313001

[jane13638-bib-0009] Cooch, E. G. (2002). Fledging size and survival in snow geese: Timing is everything (or is it?). Journal of Applied Statistics, 29, 143–162.

[jane13638-bib-0010] Cooch, E. G. , Lank, D. B. , & Cooke, F. (1996). Intraseasonal variation in the development of sexual size dimorphism in a precocial bird: Evidence from the Lesser Snow Goose. The Journal of Animal Ecology, 65(4), 439. 10.2307/5779

[jane13638-bib-0011] Cooch, E. G. , Lank, D. B. , Dzubin, A. , Rockwell, R. F. , & Cooke, F. (1991). Body size variation in Lesser Snow Geese: Environmental plasticity in gosling growth rates. Ecology, 72(2), 503–512.

[jane13638-bib-0012] Dillingham, P. W. (2010). Generation time and the maximum growth rate for populations with age‐specific fecundities and unknown juvenile survival. Ecological Modelling, 221(6), 895–899. 10.1016/j.ecolmodel.2009.12.008

[jane13638-bib-0013] Dmitriew, C. M. (2011). The evolution of growth trajectories: What limits growth rate? Biological Reviews, 86(1), 97–116. 10.1111/j.1469-185X.2010.00136.x 20394607

[jane13638-bib-0014] Dobzhansky, T. (1970). Genetics of the evolutionary process. Columbia University Press.

[jane13638-bib-0015] Doiron, M. , Gauthier, G. , & Lévesque, E. (2015). Trophic mismatch and its effects on the growth of young in an Arctic herbivore. Global Change Biology, 21(12), 4364–4376. 10.1111/gcb.13057 26235037

[jane13638-bib-0016] Eichhorn, G. , Boom, M. P. , van der Jeugd, H. P. , Mulder, A. , Wikelski, M. , Maloney, S. K. , & Goh, G. H. (2021). Circadian and seasonal patterns of body temperature in Arctic migratory and temperate non‐migratory geese. Frontiers in Ecology and Evolution, 9(June), 1–13. 10.3389/fevo.2021.699917

[jane13638-bib-0017] Eichhorn, G. , Enstipp, M. R. , Georges, J. Y. , Hasselquist, D. , & Nolet, B. A. (2019). Resting metabolic rate in migratory and non‐migratory geese following range expansion: Go south, go low. Oikos, 128(10), 1424–1434. 10.1111/oik.06468

[jane13638-bib-0018] Eichhorn, G. , Meijer, H. A. J. , Oosterbeek, K. , & Klaassen, M. (2012). Does agricultural food provide a good alternative to a natural diet for body store deposition in geese? Ecosphere, 3(4), art35. 10.1890/es11-00316.1

[jane13638-bib-0019] Eichhorn, G. , Van Der Jeugd, H. P. , Meijer, H. A. J. , & Drent, R. R. (2010). Fueling incubation: Differential use of body stores in arctic‐ and temperate‐breeding Barnacle Geese (*Branta leucopsis*). The Auk, 127(1), 162–172. 10.1525/auk.2009.09057

[jane13638-bib-0020] Endler, J. A. (1980). Natural selection on color patterns in *Poecilia reticulata* . Evolution, 34(1), 76–91. 28563214 10.1111/j.1558-5646.1980.tb04790.x

[jane13638-bib-0021] Fokkema, W. , Van der Jeugd, H. P. , Lameris, T. K. , Dokter, A. M. , Ebbinge, B. S. , de Roos, A. M. , Nolet, B. A. , Piersma, T. , & Olff, H. (2020). Ontogenetic niche shifts as a driver of seasonal migration. Oecologia, 193(2), 285–297. 10.1007/s00442-020-04682-0 32529317 PMC7320946

[jane13638-bib-0022] Garland, T. , & Adolph, S. C. (1994). Why not to do two‐species comparative studies: Limitations on inferring adaptation. Physiological Zoology, 67(4), 797–828.

[jane13638-bib-0023] Gauthier, G. , Fournier, M. , & Larochelle, J. (2006). The effect of environmental conditions on early growth in geese. Acta Zoologica Sinica, 52(1979), 670–674.

[jane13638-bib-0024] Gienapp, P. , Leimu, R. , & Merilä, J. (2007). Responses to climate change in avian migration time—Microevolution versus phenotypic plasticity. Climate Research, 35(1–2), 25–35. 10.3354/cr00712

[jane13638-bib-0025] Gompertz, B. (1825). On the nature of the function expressive of the law of human mortaliy, and on a new mode of determining the value of life contingencies. Philosophical Transactions of the Royal Society of London B: Biological Sciences, 182, 513–585. 10.1098/rstb.2014.0379 PMC4360127 25750242

[jane13638-bib-0026] Grant, P. R. , & Grant, B. R. (1995). The founding of a new population of Darwin's Finches. Evolution, 49(2), 229–240. 28565010 10.1111/j.1558-5646.1995.tb02235.x

[jane13638-bib-0027] Haywood, S. , & Perrins, C. M. (1992). Is clutch size in birds affected by environmental conditions during growth? Proceedings of the Royal Society B: Biological Sciences, 249(1325), 195–197. 10.1098/rspb.1992.0103 1360680

[jane13638-bib-0028] Hendry, A. P. , Farrugia, T. J. , & Kinnison, M. T. (2008). Human influences on rates of phenotypic change in wild animal populations. Molecular Ecology, 17(1), 20–29. 10.1111/j.1365-294X.2007.03428.x 18173498

[jane13638-bib-0029] Hendry, A. P. , & Kinnison, M. T. (1999). The pace of modern life: Measuring rates of contemporary microevolution. Evolution, 53(6), 1637–1653. 28565449 10.1111/j.1558-5646.1999.tb04550.x

[jane13638-bib-0030] Holt, R. D. , & Fryxell, J. M. (2011). Theoretical reflections on the evolution of migration. In E. J. Milner‐Gulland , J. M. Fryxell , & A. R. E. Sinclair (Eds.), Animal migration: A synthesis (pp. 17–31). Oxford University Press. 10.1093/acprof:oso/9780199568994.003.0003

[jane13638-bib-0031] Jonker, R. M. , Kraus, R. H. S. , Zhang, Q. , Van Hooft, P. , Larsson, K. , Van Der Jeugd, H. P. , Kurvers, R. H. J. M. , Van Wieren, S. E. , Loonen, M. J. J. E. , Crooijmans, R. P. M. A. , Ydenberg, R. C. , Groenen, M. A. M. , & Prins, H. H. T. (2013). Genetic consequences of breaking migratory traditions in barnacle geese *Branta leucopsis* . Molecular Ecology, 22(23), 5835–5847. 10.1111/mec.12548 24118391

[jane13638-bib-0032] Killpack, T. L. , & Karasov, W. H. (2012). Growth and development of house sparrows (*Passer domesticus*) in response to chronic food restriction throughout the nestling period. Journal of Experimental Biology, 215(11), 1806–1815. 10.1242/jeb.066316 22573759

[jane13638-bib-0033] Kim, S. Y. , Noguera, J. C. , Morales, J. , & Velando, A. (2011). Quantitative genetic evidence for trade‐off between growth and resistance to oxidative stress in a wild bird. Evolutionary Ecology, 25(2), 461–472. 10.1007/s10682-010-9426-x

[jane13638-bib-0034] Klaassen, M. (1994). Growth and energetics of tern chicks from temperate and polar environments. The Auk, 111(3), 525–544.

[jane13638-bib-0035] Kleinpeter, M. E. , & Mixner, J. P. (1947). The effect of the quantity and quality of light on the thyroid activity of the baby chick. Poultry Science, 26(5), 494–498. 10.3382/ps.0260494

[jane13638-bib-0036] Kojima, W. , Nakakura, T. , Fukuda, A. , Lin, C. P. , Harada, M. , Hashimoto, Y. , Kawachi, A. , Suhama, S. , & Yamamoto, R. (2020). Latitudinal cline of larval growth rate and its proximate mechanisms in a rhinoceros beetle. Functional Ecology, 34(8), 1577–1588. 10.1111/1365-2435.13572

[jane13638-bib-0037] Lameris, T. K. , Jochems, F. , van der Graaf, A. J. , Andersson, M. , Limpens, J. , & Nolet, B. A. (2017). Forage plants of an Arctic‐nesting herbivore show larger warming response in breeding than wintering grounds, potentially disrupting migration phenology. Ecology and Evolution, 7(8), 2652–2660. 10.1002/ece3.2859 28428856 PMC5395431

[jane13638-bib-0038] Lameris, T. K. , Van der Jeugd, H. P. , Eichhorn, G. , Dokter, A. M. , Bouten, W. , Boom, M. P. , Litvin, K. E. , Ens, B. J. , & Nolet, B. A. (2018). Arctic geese tune migration to a warming climate but still suffer from a phenological mismatch. Current Biology, 28(15), 2467–2473. 10.1016/j.cub.2018.05.077 30033332

[jane13638-bib-0039] Larsson, K. , & Forslund, P. (1991). Environmentally induced morphological variation in the barnacle goose, *Branta leucopsis* . Journal of Evolutionary Biology, 4(4), 619–636. 10.1046/j.1420-9101.1991.4040619.x

[jane13638-bib-0040] Larsson, K. , Forslund, P. , Gustafsson, L. , & Ebbinge, B. S. (1988). From the high Arctic to the Baltic: The successful establishment of a barnacle goose *Branta leucopsis* population on Gotland, Sweden. Ornis Scandinavia, 19(3), 182–189. 10.2307/3676556

[jane13638-bib-0041] Larsson, K. , van der Jeugd, H. P. , van der Veen, I. T. , & Forslund, P. (1998). Body size declines despite positive directional selection on heritable size traits in a barnacle goose population. Evolution, 52(4), 1169. 10.2307/2411246 28565228

[jane13638-bib-0042] Lindgren, B. , & Laurila, A. (2005). Proximate causes of adaptive growth rates: Growth efficiency variation among latitudinal populations of Rana temporaria. Journal of Evolutionary Biology, 18(4), 820–828. 10.1111/j.1420-9101.2004.00875.x 16033553

[jane13638-bib-0043] Lindholm, A. , Gauthier, G. , & Desrocher, A. (1994). Effects of hatch date and food supply on gosling growth in Arctic‐nesting Greater Snow Geese. The Condor, 96(4), 898–908. 10.2307/1369100

[jane13638-bib-0044] Loonen, M. J. J. E. , Bruinzeel, L. W. , Black, J. M. , & Drent, R. H. (1999). The benefit of large broods in barnacle geese: A study using natural and experimental manipulations. Journal of Animal Ecology, 68(4), 753–768. 10.1046/j.1365-2656.1999.00325.x

[jane13638-bib-0045] Madsen, J. , Cracknell, G. , & Fox, A. D. (1999). Goose populations of the Western Palearctic: A review of status and distribution. J. Madsen , G. Cracknell , & A. D. Fox (Eds.). Wetlands International Publication No. 48. Wageningen, The Netherlands: National Environmental Research insriture, Kala, Grenaavej 12, DK‐8410 Roncle, Denmark. 10.1642/0004-8038(2000)117%5B0271:r%5D2.0.co;2

[jane13638-bib-0046] Mangel, M. , & Munch, S. B. (2005). A life‐history perspective on short‐ and long‐term consequences of compensatory growth. The American Naturalist, 166(6), E155–E176. 10.1086/444439 16475079

[jane13638-bib-0047] Nolet, B. A. , Schreven, K. H. T. , Boom, M. P. , & Lameris, T. K. (2020). Contrasting effects of the onset of spring on reproductive success of Arctic‐nesting geese. The Auk, 137, 1–9. 10.1093/auk/ukz063

[jane13638-bib-0048] Owen, M. , & Black, J. M. (1989). Factors affecting the survival of Barnacle Geese on migration from the breeding grounds. Journal of Animal Ecology, 58(2), 603–617. 10.2307/4851

[jane13638-bib-0049] Pinheiro, J. , Bates, D. , DebRoy, S. , Sarkar, D. , & R Development Core Team . (2012). nlme: Linear and nonlinear mixed effects models. R package version 3.1.–104.

[jane13638-bib-0050] R Development Core Team . (2010). R: A language and environment for statistical computing. R Foundation for Statistical Computing.

[jane13638-bib-0051] Ross, M. V. , Alisauskas, R. T. , Douglas, D. C. , Kellett, D. K. , & Drake, K. L. (2018). Density‐dependent and phenological mismatch effects on growth and survival in lesser snow and Ross's goslings. Journal of Avian Biology, 49(12), 1–12. 10.1111/jav.01748

[jane13638-bib-0052] Samelius, G. , & Alisauskas, R. T. (1999). Diet and growth of glaucous gulls at a large Arctic goose colony. Canadian Journal of Zoology, 77(8), 1327–1331. 10.1139/z99-091

[jane13638-bib-0053] Schekkerman, H. , Nehls, G. , Hötker, H. , Tomkovich, P. S. , Kania, W. , Chylarecki, P. , Soloviev, M. , & Van Roomen, M. (1998). Growth of Little Stint Calidris minuta chicks on the Taimyr Peninsula, Siberia. Bird Study, 45(1), 77–84. 10.1080/00063659809461080

[jane13638-bib-0054] Schekkerman, H. , Tulp, I. , Piersma, T. , & Visser, G. H. (2003). Mechanisms promoting higher growth rate in arctic than in temperate shorebirds. Oecologia, 134(3), 332–342. 10.1007/s00442-002-1124-0 12647140

[jane13638-bib-0055] Searcy, W. A. , Peters, S. , & Nowicki, S. (2004). Effects of early nutrition on growth rate and adult size in song sparrows Melospiza melodia. Journal of Avian Biology, 35(3), 269–279. 10.1111/j.0908-8857.2004.03247.x

[jane13638-bib-0056] Sedinger, J. S. (1986). Growth and development of Canada goose goslings. The Condor, 88, 169–180. 10.2307/1368912

[jane13638-bib-0057] Sedinger, J. S. , & Flint, P. L. (1991). Growth rate is negatively correlated with hatch date in black brant. Ecology, 72(2), 496–502. 10.2307/2937190

[jane13638-bib-0058] Sedinger, J. S. , & Raveling, D. G. (1986). Timing of nesting by Canada geese in relation to the phenology and availability of their food plants. Journal of Animal Ecology, 55(3), 1083–1102. 10.2307/4435

[jane13638-bib-0059] Sofaer, H. R. , Chapman, P. L. , Sillett, T. S. , & Ghalambor, C. K. (2013). Advantages of nonlinear mixed models for fitting avian growth curves. Journal of Avian Biology, 44(5), 469–478. 10.1111/j.1600-048X.2013.05719.x

[jane13638-bib-0060] Starck, J. M. , & Ricklefs, R. E. (1998). Avian growth and development: Evolution within the altricial precocial spectrum. Oxford University Press.

[jane13638-bib-0061] Thieurmel, B. , & Elmarhraoui, A. (2019). Suncalc: Compute sun position, sunlight phases, moon position and lunar phase. R package version 0.5.0.

[jane13638-bib-0062] Tjørve, K. (2007). Does chick development relate to breeding latitude in waders and gulls? Bulletin‐Wader Study Group, April, 12–23.

[jane13638-bib-0063] Tjørve, K. M. C. , García‐Peña, G. E. , & Székely, T. (2009). Chick growth rates in Charadriiformes: Comparative analyses of breeding climate, development mode and parental care. Journal of Avian Biology, 40(5), 553–558. 10.1111/j.1600-048X.2009.04661.x

[jane13638-bib-0064] Tjørve, K. M. C. , & Tjørve, E. (2010). Shapes and functions of bird‐growth models: How to characterise chick postnatal growth. Zoology, 113(6), 326–333. 10.1016/j.zool.2010.05.003 21115290

[jane13638-bib-0065] Tjørve, K. M. C. , & Tjørve, E. (2017). The use of Gompertz models in growth analyses, and new Gompertz‐model approach: An addition to the Unified‐Richards family. PLoS ONE, 12(6), 1–17. 10.1371/journal.pone.0178691 PMC5459448 28582419

[jane13638-bib-0066] Tomotani, B. M. , Gienapp, P. , Beersma, D. G. M. , & Visser, M. E. (2016). Climate change relaxes the time constraints for late‐born offspring in a long‐distance migrant. Proceedings of the Royal Society B: Biological Sciences, 283(1839), 20161366. 10.1098/rspb.2016.1366 PMC5046899 27655765

[jane13638-bib-0072] Tulp, I. (1998). Reproductie van strandplevieren Charadrius alexandrinus en bontbekplevieren Charadrius hiaticula op Terschelling, Griend en Vlieland in 1997. Limosa, 71, 109–120.

[jane13638-bib-0067] Van der Graaf, A. J. , Stahl, J. , Drent, R. H. , Klimkowska, A. , & Bakker, J. P. (2006). Surfing on a green wave—How plant growth drives spring migration in the barnacle goose *Branta leucopsis* . Ardea, 94(3), 567–577.

[jane13638-bib-0068] Van der Jeugd, H. P. , Eichhorn, G. , Litvin, K. E. , Stahl, J. , Larsson, K. , Van Der Graaf, A. J. , & Drent, R. H. (2009). Keeping up with early springs: Rapid range expansion in an avian herbivore incurs a mismatch between reproductive timing and food supply. Global Change Biology, 15(5), 1057–1071. 10.1111/j.1365-2486.2008.01804.x

[jane13638-bib-0069] Van der Jeugd, H. P. , Gurtovaya, E. , Eichhorn, G. , Litvin, K. Y. , Mineev, O. Y. , & van Eerden, M. (2003). Breeding barnacle geese in Kolokolkova Bay, Russia: Number of breeding pairs, reproductive success and morphology. Polar Biology, 26(11), 700–706. 10.1007/s00300-003-0535-7

[jane13638-bib-0070] Van der Jeugd, H. P. , & Larsson, K. (1998). Pre‐breeding survival of barnacle geese *Branta leucopsis* in relation to fledgling characteristics. Journal of Animal Ecology, 67(6), 953–966. 10.1046/j.1365-2656.1998.6760953.x 26412374

[jane13638-bib-0071] Wineland, M. J. (2002). Fundamentals of managing light for poultry. In D. D. Bell & W. D. Jr. Weaver (Eds.), Commercial chicken meat and egg production (5th ed.). Springer. 10.1007/978-1-4615-0811-3_10

