# Supplementary material for: Postnatal growth rate varies with latitude in range‐expanding geese: The role of plasticity and day length
Source: J Anim Ecol. 2021 Nov 28;91(2):417–27. doi: 10.1111/1365-2656.13638 (PMC9300058; doi:10.1111/1365-2656.13638)
Supplement: Supplementary file 1 — Supplementary Material [file JANE-91-417-s001.docx]

**Supplementary information**

**Methods**

*Catching*

Geese moult their flight feathers simultaneously, and cannot fly for a period of a few weeks shortly after incubation when they accompany their growing, self-feeding goslings (Owen, 1980). Goslings and their parents were captured and measured during the adults´ wing moult period, when goslings were still flightless and adults had not regained flight capacity.

*Gosling age determination*

Gosling age was determined by two methods: 1) In the Barents Sea and North Sea population, goslings were individually marked with a tag attached to the foot web at the moment of hatching or immediately thereafter while still in the nest. Upon recapture before fledging (when the web tag was removed, and goslings received individually inscribed coloured leg rings) gosling age was determined as the difference in days between day of recapture and day of hatch. 2) In all populations, family relationships were determined by observing colour-ringed parents with their newly colour-ringed young (including goslings without a web tag) using spotting scopes. The age of goslings captured without web tag was thereafter estimated as the difference in days between day of capture and day when their marked parents´ brood hatched.

In the Baltic Sea population hatching dates of broods of marked pairs were determined each year by either: 1) direct observations of pairs with young leaving their nest, or 2) by estimating the age of newly-hatched young observed at a distance on grazing grounds, and then backdating. To estimate the age of newly-hatched young we compared tarsus length, neck length in relation to head length, and overall size of young, with those of the young whose age was determined by method 1. In the years 1984 to 1989 when method 2 was evaluated, the mean estimated age of goslings used to determine hatching dates of broods of marked pairs by method 2 was 3.7 days (n = 818, sd = 2.8). The mean deviation of the age estimates from the age determined by method 1 was +0.15 days (n = 121, sd = 1.67) (Larsson and Forslund 1991).

*Setting asymptotic values for the growth models*

We used fixed asymptotic values in the Gompertz growth models. Asymptotic values were based on measurements of adult geese caught during moult in the three study areas. Adult tarsus and head length did not differ between populations (F_2, 4836_ = 2.912, p = 0.055; F_2, 5838_ = 1.249, p = 0.287). Differences were significant for body mass (F_2, 6095_ = 6.159, p < 0.01), but the effect size was small, with differences less than 35 g. Moreover, body mass is variable due to deposition and depletion of body stores. Therefore, we decided to use adult size and body mass averaged over all three populations instead of using population-specific asymptotic values.

*Population mean hatch date*

We calculated the mean hatch date for every year in the study period for all three of the study colonies. The mean colony hatch date is based on directly observed hatch dates in the Baltic Sea population and is calculated for the North Sea population and Barents Sea population based on the lay dates established during nest monitoring (first egg date + 30 = hatch date; (Van der Jeugd *et al.*, 2009)). The number of nests in the study colonies fluctuated over the study period. Median number of nests (and range) was 390 (188-699), 1640 (630-2450) and 229 (136-516) for the Barents Sea, Baltic Sea and North Sea colonies respectively.

*GLMM analysis on the residuals of a non-random Gompertz curve*

We analysed our data by using GLMM’s on the residuals of non-random Gompertz models for males and females. These models followed expression [1], with fixed initial and asymptotic sizes for males and females, so without a population effect and without random effects for cohort and nestID. In the GLMM’s, the residual values of the non-random Gompertz model were used as dependent variable. Population was included as a fixed effect, and relative hatch date was included as covariate. Random effects were included for nestID nested in cohort, allowing for different slopes. We modelled body mass and structural size as a function of age in days as well as daylight experienced. We compared models with and without an effect for population by using AIC, selecting for the most parsimonious model.

**Tables and figures**


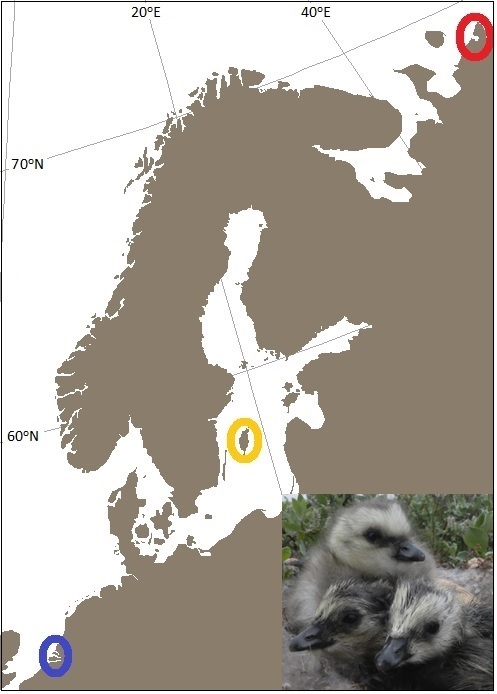


Figure S 1: Map showing locations of the study colonies. The Barents Sea colony at Kolokolkova Bay (red circle), the Baltic Sea colony on Gotland (yellow circle) and the North Sea colony in the Dutch delta (blue circle).


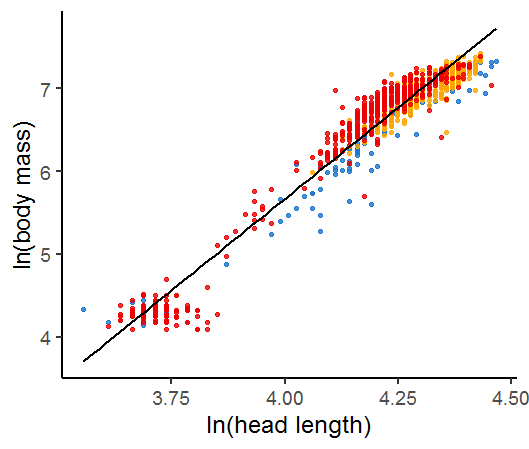


*Figure S 2: Correlation between ln(head length) and ln(body mass) for goslings of various age of the Barents Sea (red), Baltic Sea (yellow) and North Sea population (blue). The black line represents the linear relationship (y = -12.01 + 4.42x; R^2^ = 0.94).*


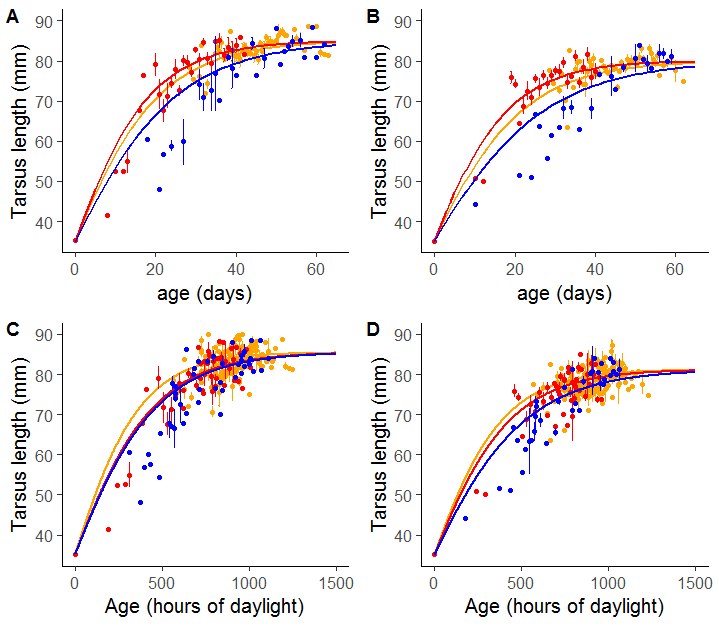


*Figure S 3: Gompertz growth models for tarsus length in relation to age in days (panel A: males, panel B: females) and hours of daylight experienced by each gosling (panel C: males, panel D: females). The Barents Sea population is shown in red, the Baltic population in yellow and the North Sea population in blue. Data points show daily means with the corresponding standard error.*


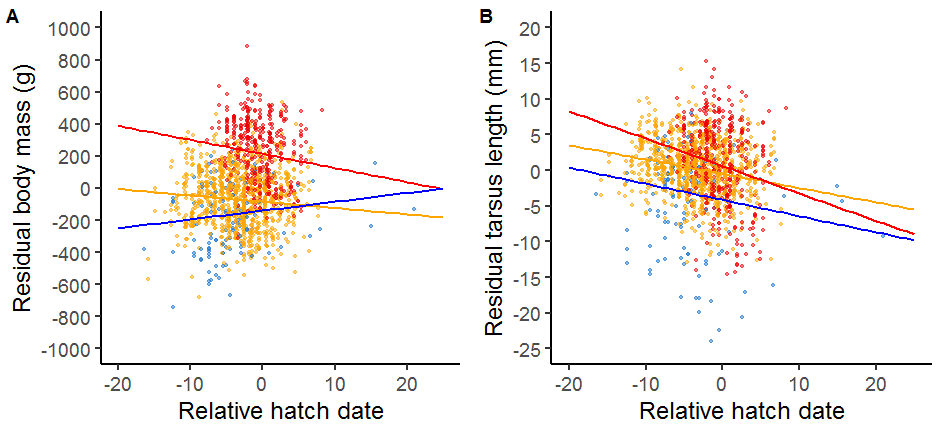


Figure S 4: The relationship between relative hatch date and residual tarsus length extracted from the non-random Gompertz growth models. The Barents Sea population is shown in red, the Baltic population in yellow and the North Sea population in blue. Data points show individual residuals, lines indicate the results of the GLMM. Note that the slopes are not found to be significantly different among populations (see main text).

*Table S 1: Sample sizes of barnacle goose goslings of known age and sex for every cohort of the three study populations. Sample sizes given here can deviate slightly from the sample sizes used in the analyses since not always all biometrics were taken from each gosling.*

|  | **Baltic Sea** | | | **Barents Sea** | | | **North Sea** | | |  |
| --- | --- | --- | --- | --- | --- | --- | --- | --- | --- | --- |
| **Year** | **Females** | **Males** | **Total** | **Females** | **Males** | **Total** | **Females** | **Males** | **Total** | **Grand Total** |
| **1986** | 35 | 33 | 68 |  |  |  |  |  |  | 68 |
| **1987** | 20 | 22 | 42 |  |  |  |  |  |  | 42 |
| **1988** | 37 | 33 | 70 |  |  |  |  |  |  | 70 |
| **1989** | 18 | 24 | 42 |  |  |  |  |  |  | 42 |
| **1990** | 19 | 34 | 53 |  |  |  |  |  |  | 53 |
| **1991** | 34 | 22 | 56 |  |  |  |  |  |  | 56 |
| **1992** | 23 | 18 | 41 |  |  |  |  |  |  | 41 |
| **1993** | 48 | 48 | 96 |  |  |  |  |  |  | 96 |
| **1994** | 39 | 40 | 79 |  |  |  |  |  |  | 79 |
| **1995** | 40 | 53 | 93 |  |  |  |  |  |  | 93 |
| **1996** | 42 | 41 | 83 |  |  |  |  |  |  | 83 |
| **1997** | 37 | 35 | 72 |  |  |  |  |  |  | 72 |
| **1998** | 16 | 7 | 23 |  |  |  |  |  |  | 23 |
| **1999** | 50 | 30 | 80 |  |  |  |  |  |  | 80 |
| **2000** | 19 | 16 | 35 |  |  |  |  |  |  | 35 |
| **2003** |  |  |  | 27 | 17 | 44 |  |  |  | 44 |
| **2004** |  |  |  | 44 | 53 | 97 | 9 | 10 | 19 | 116 |
| **2005** |  |  |  | 24 | 25 | 49 | 1 | 4 | 5 | 54 |
| **2012** |  |  |  |  |  |  | 28 | 26 | 54 | 54 |
| **2013** |  |  |  | 4 | 5 | 9 |  |  |  | 9 |
| **2014** |  |  |  | 60 | 70 | 130 |  |  |  | 130 |
| **2015** |  |  |  | 24 | 39 | 63 | 9 | 12 | 21 | 84 |
| **2018** |  |  |  |  |  |  | 6 | 11 | 17 | 17 |
| **Combined** | 477 | 456 | 933 | 183 | 209 | 392 | 53 | 63 | 116 | 1441 |

*Table S 2: Overview of the initial values (size at hatching, I) and asymptotic values (adult size, A) used in the Gompertz equations for body mass, tarsus length and head length, for male and female barnacle geese respectively (mean ± SE).*

|  | **Males** | | **Females** | |
| --- | --- | --- | --- | --- |
| **Biometric measure** | ***I*** | ***A*** | ***I*** | ***A*** |
| Body mass (g) | 72 ± 0.51 | 1869 ± 3.16 | 72 ± 0.51 | 1615 ± 2.65 |
| Tarsus Length (mm) | 35.1 ± 0.43 | 85 ± 0.05 | 35.1 ± 0.43 | 80 ± 0.23 |
| Head Length (mm) | 41 ± 0.16 | 85 ± 0.10 | 41 ± 0.16 | 81 ± 0.28 |

*Table S 3: Model comparisons of GLMM’s on the residuals of non-random Gompertz curves with age in days and age in hours of daylight. Models include random effects for nestID, nested in cohort and hatch date as covariate (allowing for different slopes within cohorts). Comparisons on the inclusion of a fixed population effect are made for males and females separately, selecting fort the most parsimonious model based on AIC (indicated by the *).*

| **Model** | **age parameter** | **Sex** | **K** | **AICc** | **∆AICc** | **LL** |
| --- | --- | --- | --- | --- | --- | --- |
| residual body mass ~ Population* | days | Males | 11.00 | 9319.59 | 0.00 | -4648.61 |
| residual body mass ~ int | days | Males | 9.00 | 9341.73 | 22.14 | -4661.74 |
| residual body mass ~ Population* | days | Females | 11.00 | 9017.45 | 0.00 | -4497.53 |
| residual body mass ~ int | days | Females | 9.00 | 9043.43 | 25.98 | -4512.59 |
| residual body mass ~ Population* | daylight | Males | 11.00 | 9320.23 | 0.00 | -4648.93 |
| residual body mass ~ int | daylight | Males | 9.00 | 9324.25 | 4.02 | -4653.00 |
| residual body mass ~ Population* | daylight | Females | 11.00 | 9018.87 | 0.00 | -4498.24 |
| residual body mass ~ int | daylight | Females | 9.00 | 9026.37 | 7.50 | -4504.06 |
| residual head length ~ Population* | days | Males | 11.00 | 3735.37 | 0.00 | -1856.50 |
| residual head length ~ int | days | Males | 9.00 | 3742.82 | 7.44 | -1862.28 |
| residual head length ~ Population* | days | Females | 11.00 | 3630.36 | 0.00 | -1803.99 |
| residual head length ~ int | days | Females | 9.00 | 3638.22 | 7.86 | -1809.98 |
| residual head length ~ Population | daylight | Males | 11.00 | 3732.49 | 3.25 | -1855.06 |
| residual head length ~ int* | daylight | Males | 9.00 | 3729.23 | 0.00 | -1855.49 |
| residual head length ~ Population | daylight | Females | 11.00 | 3626.07 | 2.46 | -1801.85 |
| residual head length ~ int* | daylight | Females | 9.00 | 3623.61 | 0.00 | -1802.68 |
| residual tarsus length ~ Population* | days | Males | 11.00 | 4082.90 | 0.00 | -2030.26 |
| residual tarsus length ~ int | days | Males | 9.00 | 4091.02 | 8.13 | -2036.39 |
| residual tarsus length ~ Population* | days | Females | 11.00 | 3969.73 | 0.00 | -1973.68 |
| residual tarsus length ~ int | days | Females | 9.00 | 3977.53 | 7.79 | -1979.63 |
| residual tarsus length ~ Population | daylight | Males | 11.00 | 4062.88 | 0.00 | -2020.26 |
| residual tarsus length ~ int* | daylight | Males | 9.00 | 4064.38 | 1.49 | -2023.06 |
| residual tarsus length ~ Population | daylight | Females | 11.00 | 3944.05 | 0.00 | -1960.83 |
| residual tarsus length ~ int* | daylight | Females | 9.00 | 3945.32 | 1.28 | -1963.53 |

*Table S 4 Overview of the Gompertz growth coefficients (K), breeding latitude and body mass (g) of species used in the species comparison (fig S 2).*

| **Species** | **Latitude** | **Body mass** | **K** | **Reference** |
| --- | --- | --- | --- | --- |
| Calidris alba | 76°N | 81 | 0.085 | Tjørve 2007 |
| Calidris alpina | 61°N | 40 | 0.138 | Tjørve 2007 |
| Calidris bairdii | 72°N | 48 | 0.12 | Tjørve 2007 |
| Calidris canutus | 75°N | 120 | 0.163 | Tjørve 2007 |
| Calidris ferruginea | 75°N | 52 | 0.214 | Tjørve 2007 |
| Calidris fuscicollis | 76°N | 37 | 0.158 | Tjørve 2007 |
| Calidris melanotos | 72°N | 60 | 0.106 | Tjørve 2007 |
| Calidris minuta | 72°N | 26.6 | 0.159 | Tjørve 2007 |
| Calidris minuta | 74°N | 26.7 | 0.191 | Tjørve 2007 |
| Calidris pusilla | 72°N | 26 | 0.151 | Tjørve 2007 |
| Charadrius dubius | 48°N | 41 | 0.099 | Tjørve 2007 |
| Charadrius hiaticula | 53°N | 67 | 0.058 | Tulp 1998 |
| Charadrius hiaticula | 56°N | 67 | 0.108 | Tjørve 2007 |
| Charadrius hiaticula | 72°N | 67 | 0.108 | Tjørve 2007 |
| Charadrius morinellus | 57°N | 108 | 0.08 | Tjørve 2007 |
| Limosa limosa | 53°N | 273 | 0.085 | Tjørve 2007 |
| Numenius arquata | 53°N | 990 | 0.051 | Tjørve 2007 |
| Philomachus pugnax | 53°N | 125 | 0.092 | Tjørve 2007 |
| Tringa totanus | 53°N | 137 | 0.07 | Tjørve 2007 |
| Vanellus vanellus | 53°N | 236 | 0.054 | Tjørve 2007 |
| Vanellus vanellus | 56°N | 255 | 0.079 | Tjørve 2007 |
| Anser indicus | 43°N | 2600 | 0.030 | Starck & Ricklefs 1998 |
| Branta bernicla nigrans | 61°N | 1237 | 0.055 | Sedinger & Flint 1991 |
| Branta canadensis minima | 61°N | 1400 | 0.074 | Sedinger 1986 |
| Chen caerulescens atlantica | 71°N | 2500 | 0.062 | Lesage & Gauthier 1997 |
| Cygnus cygnus | 65°N | 9000 | 0.032 | Knudsen et al. 2002 |
| Cygnus olor | 54°N | 12000 | 0.022 | De Leeuw & Beekman 1991 |
| Branta leucopsis (North Sea) | 51°N | 1800 | 0.037 | This study |
| Branta leucopsis (Baltic Sea) | 57°N | 1800 | 0.041 | This study |
| Branta leucopsis (Barents Sea) | 68°N | 1800 | 0.0575 | This study |
